# Acceptability and Feasibility of an Islamic Faith-Based Smoking Cessation Intervention Delivered via WhatsApp for Muslim Smokers in Saudi Arabia: Pilot Randomized Controlled Trial

**DOI:** 10.2196/88796

**Published:** 2026-10-15

**Authors:** Abdullah Kalantan, Samantha Marsh, Sarah S Monshi, Amer Siddiq Amer Nordin, Chris Bullen

**Affiliations:** 1 Department of General Practice and Primary Healthcare, School of Population Health, Faculty of Medical and Health Sciences, University of Auckland, Level 2, Building 507, 22-30 Park Ave, Grafton Campus, Auckland, 1142, New Zealand, 64 273393244; 2 Department of Health Administration and Hospitals, College of Public Health and Health Informatics, Umm al-Qura University, Mecca, Saudi Arabia; 3 Department of Psychological Medicine, Faculty of Medicine, Universiti Malaya, Malaysia and University Malaya Centre of Addiction Sciences (UMCAS), University of Malaya, Kuala Lumpur, Malaysia

**Keywords:** smoking cessation, faith, religion, whatsApp messaging, behavioral intervention, Saudi Arabia

## Abstract

**Background:**

Smoking is common in Saudi Arabia, particularly among men. Religion plays a protective role against smoking and serves as a motivator for quitting. Faith-based interventions have shown positive effects in supporting smoking cessation among Muslims who smoke; however, few studies have tested their acceptability when delivered via mobile phone messaging.

**Objective:**

The aim of this pilot randomized controlled trial was to evaluate the feasibility and acceptability, as well as the preliminary effectiveness of an Islamic-based smoking cessation intervention delivered via WhatsApp messaging in Saudi Arabia.

**Methods:**

This study was a 2-arm randomized controlled trial involving adult Muslim smokers who used cigarettes, waterpipe, or both. Participants were recruited from the Kafa organization antismoking clinic in Saudi Arabia. Participants were randomized in a 1:1 ratio to either the intervention or control group. The intervention messages were codeveloped with religious leaders and informed by formative research with Muslim current and former smokers. The intervention group received both religious and smoking-related health messages, whereas the control group received smoking-related health messages only. The messages were manually delivered daily by trained research assistants. The intervention lasted for 21 days, during which participants received 2 messages per day, one in the morning and one in the evening. The primary outcomes were the feasibility and acceptability of the intervention.

**Results:**

A total of 34 participants were recruited, resulting in a recruitment rate of 68% (34/50). The overall retention rate was 64.7% (22/34). Both groups received 44 WhatsApp messages over 21 days. Message engagement was high, with 88.2% (15/17) of participants in the intervention group and 82.4% (14/17) in the control group, reporting that they read the daily messages. The intervention was acceptable and was perceived as helpful in supporting smoking cessation. Complete abstinence from both cigarettes and waterpipe was slightly higher in the intervention group (4/17, 23.5%) than in the control group (3/17, 17.6%). Cigarette abstinence was also modestly higher in the intervention group than in the control group (5/16, 31.3% vs 3/16, 18.8%). Among 8 waterpipe users at baseline, 2 reported waterpipe abstinence at follow-up, both in the intervention group. No statistical comparisons of smoking cessation outcomes between the groups were conducted because the study focused on feasibility and included a small sample size. These findings were descriptive and should be considered exploratory rather than evidence of intervention effectiveness.

**Conclusions:**

This Islamic faith-based intervention, delivered via WhatsApp messaging, was acceptable and may be feasible for a full-scale trial, provided that recruitment, participant retention, and follow-up data collection are improved. The pilot study provides important lessons for intervention design and implementation of a future adequately powered trial.

## Introduction

Tobacco use is a major public health concern worldwide. Every year, tobacco kills more than 7 million people globally, including an estimated 1.6 million nonsmokers who are exposed to second-hand smoke [1]. Smoking prevalence is high among Muslim populations. The prevalence of smoking is particularly high in Muslim-majority countries such as Saudi Arabia, Egypt, Jordan, and Indonesia [2]. Furthermore, smoking rates are considerably higher among Muslim minorities living in Western countries compared to the general population in England [3] and the United States [4].

Saudi Arabia had an adult smoking prevalence of 16.5% in 2022 [5]. A recent systematic review, in 2023, found that smoking prevalence was high among Saudi school students (estimates ranged from 10.0% to 21.7%), university students (range 11.1%‐25.3%), and the general population (range 19.6%‐23.5%), particularly among men [6]. Although smoking is socially and religiously discouraged in Saudi Arabia, particularly among women [7], evidence indicates that smoking rates remain high in Saudi Arabia [8]. The most common form of tobacco use in Saudi Arabia is cigarette smoking, followed by waterpipe use [6,9].

Studies investigating the reasons for smoking initiation and continuation in Saudi Arabia highlight factors such as social influence, entertainment, curiosity, stress relief, socializing, and the ready accessibility of tobacco products [10-13]. Among waterpipe users, the perception that it is less harmful and more socially acceptable than cigarette smoking has contributed to its growing popularity [14].

Tobacco control policies and initiatives in line with the World Health Organization’s (WHO) MPOWER measures were implemented in Saudi Arabia, including increased sales taxes, anti-smoking awareness campaigns, and smoking cessation services [15], but the use is low among some subgroups [16]. Despite efforts to reduce tobacco consumption, recent cross-sectional evidence suggests an increase in smoking prevalence in Saudi Arabia [17,18]. For these reasons, innovative approaches to reach and support people to quit smoking are needed.

Faith-based approaches to health promotion can foster trust and connection, and religious content can enhance participants’ motivation, engagement, and sense of belonging [19]. Embedding health interventions within an Islamic context can enhance their relevance and acceptability among Muslim communities, and involving Muslim religious leaders (*Imams*) plays a critical role in promoting health among Muslim populations [20]. Adherence to religious values and practices acts as a protective factor against smoking [9,21,22]. The Saudi constitution is founded on the *Quran* and the *Sunnah*, with *Sharia* law forming the basis of the legal system. Religion has been central to shaping individuals’ personal and collective identities and to consolidating national values in Saudi Arabia [23].

Studies conducted in Saudi Arabia have also shown that religion is a common motivator to quit smoking [7,24]. Furthermore, a recent study reported that Saudi Muslims who smoke were motivated to quit smoking during religious occasions such as Ramadan [25]. These findings highlight the importance of developing and evaluating faith-based approaches to smoking cessation.

A challenge with all smoking cessation interventions is how to reach people to support them to quit successfully. Text messaging–based smoking cessation interventions are now well-established and have been shown to be effective [26,27]. The success of text messaging in delivering smoking cessation intervention is due to its wide reach, low cost, high-read rates, and flexibility to be tailored and personalized [28]. WhatsApp is the most commonly used social media platform among the Saudi population [29]. It has been shown to be an effective and acceptable method for supporting smoking cessation in other countries and settings [30,31].

This pilot randomized controlled trial (RCT) aimed to assess the feasibility, acceptability, and preliminary effectiveness of a faith-based smoking cessation intervention delivered via WhatsApp messages. The popularity and accessibility of WhatsApp in Saudi Arabia make it a culturally and contextually appropriate medium for delivering behavioral and faith-based health interventions. The results of this study will inform the design and conduct of a future, more definitive, adequately powered RCT to assess the effectiveness of the intervention on smoking cessation.

## Methods

### Trial Design

A single-blind, parallel, two-arm, pilot RCT to test the feasibility, acceptability, and preliminary effectiveness of the Islamic faith-based smoking cessation intervention delivered via the social media platform WhatsApp. The intervention was delivered over a 21-day period (from July 4 to July 24, 2025) to keep it feasible and reduce participant burden, while still providing intensive support during the early weeks of abstinence, when relapses are most likely to occur [32].

### Participants

Potential participants were identified and recruited by 2 trained research assistants at the Kafa organization’s clinic in Makkah, Saudi Arabia. Kafa is a nongovernmental organization dedicated to raising awareness about the harms of smoking and substance abuse and providing both pharmacological and behavioral treatments. Recruitment took place between May 1 and June 30, 2025 (2-month period). Interested participants were provided with the participant information sheet and consent form. The research assistants briefly explained the trial protocols to participants and answered their questions about the study. Eligible participants who agreed to take part provided informed consent.

The inclusion criteria were adults aged 18 years or older who currently smoke cigarettes, waterpipe, or both; self-identify as Muslims; are residents of Saudi Arabia; own a smartphone with the WhatsApp application; and can read and understand Arabic. Formal digital literacy was not assessed. The study included participants regardless of whether they were willing to quit or not. Participants were ineligible if they were receiving another behavioral or pharmaceutical therapy to help them quit smoking or were diagnosed with mental health disorders for which they were receiving medical treatments from a health professional.

This trial focused on combustible tobacco products, including hand-rolled and factory-made cigarettes and waterpipes. A current cigarette smoker was defined as an adult who has smoked at least 100 cigarettes in their lifetime and who currently smokes cigarettes [33]. Similarly, a current waterpipe smoker was defined as an adult who has smoked using waterpipes at least once in their lifetime and who currently smokes waterpipes.

### Sample Size

An a priori sample size calculation is not required for a pilot RCT, as it is not designed to estimate outcomes or conduct hypothesis testing; however, the sample needs to be large enough to provide useful information about feasibility outcomes [34]. Hence, no formal sample size estimation was performed. Based on recommendations for pilot RCTs, as well as time and resource constraints, a sample of 50 participants (25 participants per arm) was deemed appropriate [35].

### Randomization and Allocation

Eligible participants who provided consent were randomly assigned to either the intervention group or the control group using simple randomization in a 1:1 allocation ratio. For this purpose, a randomization table was generated by an independent researcher using the REDCap software (Vanderbilt University). AK assigned participants to their groups after enrollment. Allocation was concealed from research assistants who were informed of participants’ group after enrollment and just before administering the intervention.

### Blinding

Considering the nature of the intervention, blinding of participants was not possible: participants in the intervention group were aware that they were receiving a combination of religious and smoking-related health messages, whereas participants in the control group knew that they were receiving smoking-related health messages only. However, the outcome assessors were blinded to treatment allocation to minimize the risk of biased assessment of the intervention’s effects. The lead author (AK) was not involved in delivering the intervention, which was administered by a research assistant in Saudi Arabia. All datasets were coded by participants’ ID number only, and the author analyzed the data while remaining blind to group allocation.

### Faith-Based Intervention

The faith-based intervention integrated both Islamic-based smoking cessation messages and smoking-related health messages (Multimedia Appendix 1). The religious messages, developed in conjunction with *Imams*, were derived from the *Quran* (Islam’s Holy Book) and *Hadith* (the sayings and actions of the Prophet Mohammed). The smoking-related health messages provided behavioral strategies for quitting smoking, information on the harms of smoking and the benefits of cessation, motivational statements, and details about available cessation resources (eg, Quitline phone number). These smoking-related health messages were adapted from the existing text message library of the STOMP smoking cessation program [36] and informed by qualitative individual interviews with Muslim current and former smokers.

The intervention was pretested during Ramadan 2024 (March 10 to April 9) and across 4 weeks after Ramadan with 4 Muslim adults. The intervention underwent major modifications during the development phase, including changes to message content, delivery features, message timing and frequency, and overall intervention duration. The intervention content and delivery schedule remained unchanged throughout the implementation period.

A total of 44 messages were delivered over 21 days (July 4 to July 24, 2025). These comprised 1 welcome message sent at enrollment, 2 messages delivered daily (one in the morning and one in the evening), and 1 farewell message sent at the conclusion of the intervention. All messages were written in Arabic and were under 500 words. Participants were not required to set a quit date. Two trained research assistants with experience in smoking cessation treatment manually delivered the daily messages via the Kafa organization’s WhatsApp channel, rather than using an automated system.

The intervention group received both the Islamic-based smoking cessation messages and smoking-related health messages. On the other hand, the control group received smoking-related health messages without religious content. To maintain equivalent exposure intensity between groups, participants in the control group received a greater number of smoking-related health messages than those in the intervention group.

### Data Collection

Baseline and follow-up data were collected via internet-based questionnaires using the REDCap software. Participants were not required to visit a clinic in person for data collection. The research assistants distributed the baseline and follow-up questionnaire links to participants via WhatsApp.

At baseline, demographic information was collected, including age, gender, ethnicity, highest level of education, employment status, marital status, and types of tobacco products smoked. The number of cigarettes and waterpipe sessions smoked per day, nicotine dependence, religiosity, motivation to stop smoking (MTSS), previous quit attempts, and use of e-cigarettes were also measured at baseline. Nicotine dependence was measured using 2 different instruments: one for cigarette smoking and another for waterpipe smoking. For cigarette smokers, nicotine dependence was measured by the Fagerström Test for Nicotine Dependence (FTND) [37]. Dependence for waterpipe smokers was measured by the 11-item Lebanon Waterpipe Dependence Scale (LWDS-11) [38]. Religiosity was measured using the Duke University Religion Index (DUREL) [39], assessing 3 dimensions of religiosity: organizational religious activity (ORA), nonorganizational religious activity (NORA), and intrinsic religiosity (IR).

Follow-up data collection was administered at the end of the intervention (day 21) to collect data on smoking abstinence, MTSS, quit attempts, and intervention acceptability.

### Primary Outcome Measures

The primary outcomes were measures of feasibility and acceptability. Feasibility was assessed using recruitment, retention, and adherence to protocols. No formal a priori progression criteria or feasibility thresholds were specified for recruitment or retention. However, these outcomes were interpreted in relation to previous studies to provide a contextual assessment of feasibility [40].

Retention was measured as the percentage of participants who completed the follow-up questionnaire (day 21) out of the total number enrolled in the trial. Adherence to the text messages was measured by sending participants a daily follow-up message asking whether they had read the messages. This follow-up message was sent late in the evening after the delivery of the second daily message. Participants were instructed to respond either by replying “yes” to the message or by using a thumbs-up emoji to indicate that they had read it. Participants were classified into 3 groups: daily readers (who read messages every day during the 21-d intervention period), partial readers (who read messages on some but not all days), and nonreaders (who did not read any messages at all). Intervention acceptability was measured using the generic Theoretical Framework of Acceptability (TFA)–based questionnaire [41]. The TFA-based questionnaire consisted of 9 items: one item assessing each of the TFA constructs (affective attitude, burden, perceived effectiveness, intervention coherence, self-efficacy, and opportunity cost), 2 items assessing ethicality, and 1 item assessing the general acceptability item.

The TFA-based questionnaire can be adapted to assess the acceptability of different health care interventions by specifying either the intervention itself or the behavior required to engage with it [41]. We adapted the TFA-based questionnaire to evaluate the acceptability of the Islamic faith-based smoking cessation intervention. The questionnaire included items assessing the overall acceptability (eg, *How acceptable was the Islamic faith-based intervention for quitting smoking to you?*), affective attitude (eg, *Did you like or dislike the Islamic faith-messages?*), burden (eg, *How much effort did it take you to read and engage with the messages?*), perceived effectiveness (eg, *The messages helped me to either quit smoking or reduce the amount I smoke*), and opportunity cost (eg, *Taking part in the study and reading the daily messages conflicted with my other commitments and priorities*). Additionally, participants were asked open-ended questions to provide feedback on the strengths and limitations of the intervention and offer recommendations for improvement.

### Secondary Outcome Measures

Smoking abstinence and quit attempts were measured as indicators of cessation progress. Motivation to quit smoking was measured to assess how religious messages may enhance motivation to quit.

Smoking abstinence was measured by self-report, with 7-day point prevalence abstinence defined as complete cessation, not even a single puff, during the last 7 days [42]. Motivation to quit was assessed at baseline and at the end of the intervention (day 21) using the 7-level, single-item MTSS instrument [43]. A serious quit attempt was defined as a period of intentional abstinence from smoking lasting at least 24 hours [42]. At baseline, quit attempts were measured by asking participants if they had made a serious quit attempt to quit smoking in the past 30 days. At follow-up, quit attempts were measured by asking participants if they had made a serious quit attempt to stop smoking that lasted at least 24 hours in the past 21 days (during the intervention period).

### Data Analysis

The intention-to-treat method was used for data analysis [44], whereby respondents were included in the analysis according to their original responses, regardless of whether they completed the study. For smoking abstinence outcomes, participants with missing follow-up data were treated as continuing to smoke. For cigarette and waterpipe use outcomes, missing follow-up data were handled by carrying forward baseline values, assuming no change from baseline. For MTSS and quit attempts, baseline responses were carried forward for participants with missing follow-up data. This approach maintains the benefits of randomization while minimizing the risk of bias in estimating the effects of intervention. However, as this was a feasibility pilot study, statistical precision for effectiveness outcomes was limited. Descriptive statistics were used to describe baseline and follow-up data, including key feasibility and acceptability outcomes. Bivariate analyses were conducted to explore the association between exposure to intervention messages and smoking abstinence. In addition, multivariable binary logistic regression models were used to explore the associations between each DUREL subscale and smoking abstinence, adjusting for treatment groups. Analyses were conducted using R software (version 4.6.1; R Foundation for Statistical Computing). Baseline demographic data were compared to determine the balance of the randomization.

### Ethical Considerations

The trial was approved by the Auckland Health Research Ethics Committee (number AH27150) in New Zealand and the Biomedical Research Ethics Committee at Umm Al-Qura University (number HAPO-02-K-012) in Saudi Arabia. A collaboration and nondisclosure agreement was signed with the Kafa organization. This trial is registered at the Australian New Zealand Clinical Trials Registry (ACTRN 12625001413415). The trial was retrospectively registered on December 16, 2025, after the first participant was enrolled on May 1, 2025. Prospective registration was not undertaken because the research team understood that registration was not required for this pilot trial conducted as part of a PhD research project.

Eligible participants were screened by a research assistant. All participants provided informed consent, which was obtained online via REDCap. Participants were offered a shopping voucher worth US $25 in appreciation of their participation. Baseline and follow-up data were collected and managed using REDCap. All data were deidentified prior to analysis to ensure participants’ privacy and confidentiality. All research data were securely stored on a password-protected University of Auckland–managed storage service (ie, research drive) on a password-protected computer. Only the principal investigator (AK) had access to the data.

## Results

### Participant Engagement and Feasibility Outcomes

Participant recruitment, randomization, follow-up, and analysis are presented in the CONSORT (Consolidated Standards of Reporting Trials) flow diagram in Figure 1. The target sample size was 25 participants per group; however, we did not achieve the target number by the end of the recruitment period (2 mo). In this study, a total of 43 participants were assessed for eligibility, of whom 34 (68% of the target of 50) participants met the inclusion criteria and were randomized. Participants were randomly assigned to the intervention group (n=17) or control group (n=17). All randomized participants completed baseline assessment (N=34). At the end of the intervention (day 21), 22 participants completed the follow-up survey, corresponding to an overall retention rate of 64.7% (22/34). Retention was equal in both groups, with 11 participants from the intervention group and 11 from the control group. Among the 22 respondents, 7 (31.8%) participants submitted incomplete survey responses. No adverse events, privacy breaches, or technical problems related to the intervention were reported during the trial.

**Figure 1.**
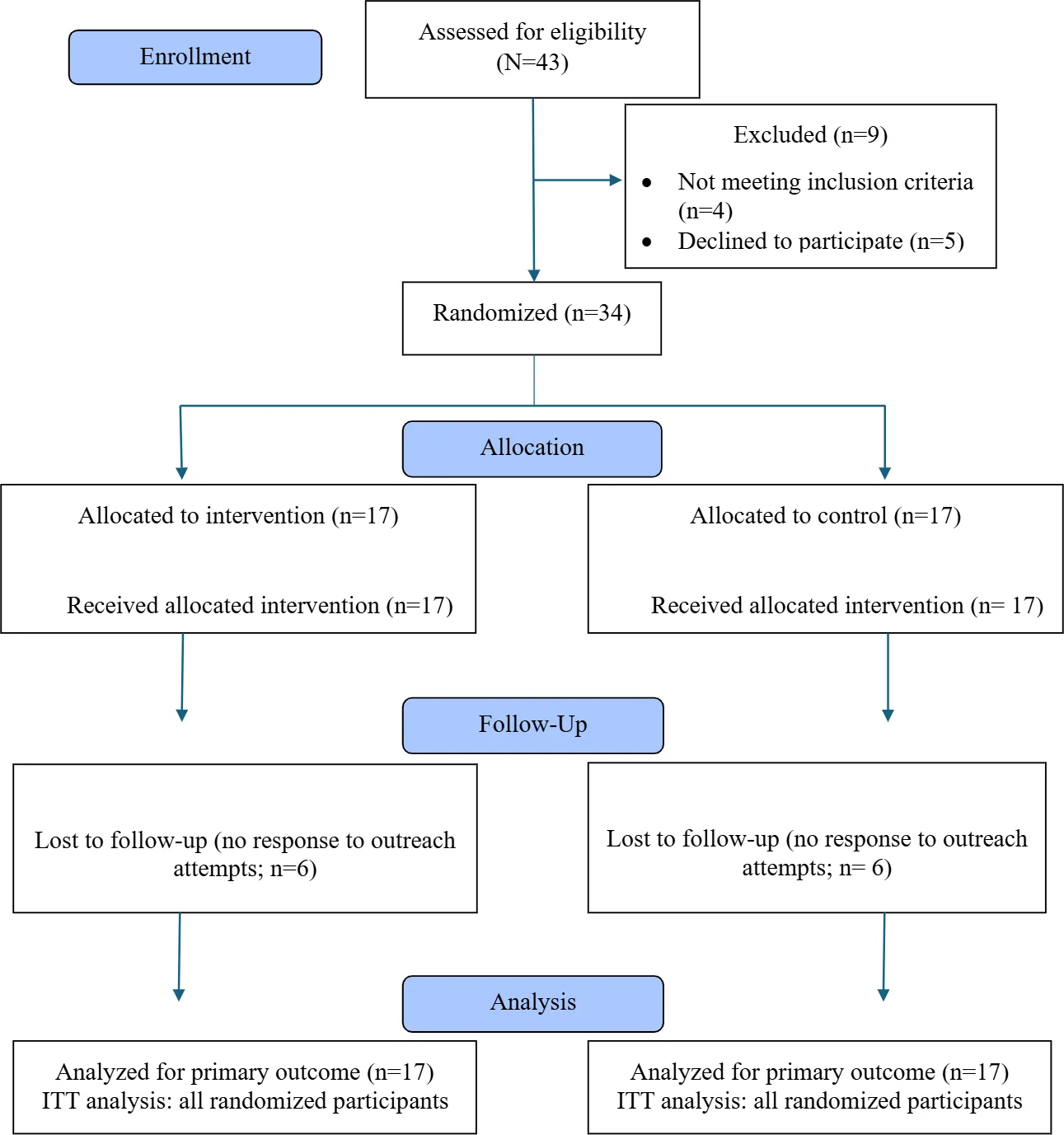
CONSORT (Consolidated Standards of Reporting Trials) flow diagram of participant enrollment, randomization, follow-up, and analyses. ITT: intention to treat.

A total of 44 WhatsApp messages were delivered to all participants in both groups over 21 days. These comprised 1 welcome message at enrollment, 42 intervention messages delivered at a rate of 2 per day (one in the morning and one in the evening), and 1 farewell message at the end of the intervention. Across the 21-day intervention period, all participants reported reading at least some of the messages. In the intervention group (n=17), 15 (88.2%) participants were daily readers and 2 (11.8%) participants were partial readers. In the control group (n=17), 14 (82.4%) participants were daily readers and 3 (17.6%) participants were partial readers. No participants in either group reported not reading any messages.

### Participant Characteristics at Baseline

The demographics of the participants are presented in Table 1. Participants were recruited from the Kafa organization’s antismoking clinics in Makkah, Saudi Arabia. The mean ages were 33.4 (SD 8.0) years in the control group and 33.7 (SD 7.8) years in the intervention group. Overall, most (32/34, 94.1%) participants were male. In both the intervention and control groups, 94.1% (16/17) of participants were men and 5.9% (1/17) were women. Regarding the education level, the highest proportion of participants (20/34, 58.8%) had a high school education or less in both groups, while 26.5% (9/34) held a bachelor’s degree and 14.7% (5/34) held a diploma. In terms of employment, the highest proportion of participants (16/34, 47.1%) were employed in the private sector, while 23.5% (8/34) were unemployed and 14.7% (5/34) were government employees. Regarding marital status, 47.1% (16/34) of participants were married in both groups.

**Table 1.** Participants’ characteristics at baseline (N=34).

Characteristics	Control (n=17)	Intervention (n=17)	Total (N=34)
Age (y), mean (SD); range	33.4 (8.0); 21‐50	33.7 (7.8); 21-44	33.6 (7.8); 21‐50
Gender, n (%)			
Male	16 (94.1)	16 (94.1)	32 (94.1)
Female	1 (5.9)	1 (5.9)	2 (5.9)
Education, n (%)			
High school or below	12 (70.6)	8 (47.1)	20 (58.8)
Diploma	2 (11.8)	3 (17.6)	5 (14.7)
Bachelor	3 (17.6)	6 (35.3)	9 (26.5)
Employment, n (%)			
Government employee	2 (11.8)	3 (17.6)	5 (14.7)
Private sector employee	10 (58.8)	6 (35.3)	16 (47.1)
Self-employed	1 (5.9)	2 (11.8)	3 (8.8)
Unemployed	2 (11.8)	6 (35.3)	8 (23.5)
Student	1 (5.9)	0 (0.0)	1 (2.9)
Retired	1 (5.9)	0 (0.0)	1 (2.9)
Marital status, n (%)			
Married	9 (52.9)	7 (41.2)	16 (47.1)
Type of tobacco used, n (%)			
Cigarettes only	13 (76.5)	13 (76.5)	26 (76.5)
Waterpipe only	1 (5.9)	1 (5.9)	2 (5.9)
Cigarettes and waterpipe	3 (17.6)	3 (17.6)	6 (17.6)
Use of e-cigarettes or vapes, n (%)			
Yes	6 (35.3)	2 (11.8)	8 (23.5)
Religiosity, mean (SD)			
ORA^a^	3.7 (2.0)	4.1 (1.8)	3.9 (1.9)
NORA^b^	5.5 (1.1)	4.8 (1.6)	5.1 (1.4)
IR^c^	4.8 (0.3)	4.9 (0.2)	4.9 (0.3)

a ORA: organizational religious activity.

b NORA: nonorganizational religious activity.

c IR: intrinsic religiosity.

Cigarette smoking was the predominant form of tobacco use (26/34, 76.5%), followed by the dual use of cigarettes and waterpipe (6/34, 17.6%). Only 2 out of 34 (5.9%) participants reported waterpipe smoking only. E-cigarette or vape use was reported by 23.5% (8/34) of participants in both groups, more commonly in the control group (6/34, 35.3%) than in the intervention group (2/34, 11.8%).

Baseline religiosity score levels were generally high across both groups, with minor differences between them. ORA scores were slightly higher in the intervention group (mean 4.1, SD 1.80) than in the control group (mean 3.7, SD 2.0). IR scores were similar across groups, with a mean of 4.9 (SD 0.2) in the intervention group and 4.8 (SD 0.3) in the control group. In contrast, private NORA scores were slightly higher in the control group (mean 5.5, SD 1.1) than in the intervention group (mean 4.8, SD 1.6).

### Nicotine Dependence at Baseline

Table 2 presents baseline nicotine dependence scores among participants with available data. The FTND was relevant to 32 participants who reported cigarette smoking at baseline; however, complete FTND data were available for 27 participants, including 14 in the control group and 13 in the intervention group. Among these participants, the FTND score was slightly higher in the control group (mean 7.0, SD 1.3) than in the intervention group (mean 6.7, SD 1.7). The scores indicate that both groups showed medium to high nicotine dependence levels at baseline.

**Table 2.** Nicotine dependence scores for cigarettes and waterpipe at baseline.

Group	Scale	Mean (SD)
Control (n=14)	FTND^a^	7.0 (1.3)
Intervention (n=13)	FTND	6.7 (1.7)
Control (n=3)	LWDS-11^b^	14.7 (5.6)
Intervention (n=2)	LWDS-11	18.5 (7.8)

a FTND: Fagerström Test for Nicotine Dependence.

b LWDS: Lebanon Waterpipe Dependence Scale.

The LWDS-11 was applicable to 8 participants who reported waterpipe smoking at baseline; however, only 5 participants completed the LWDS-11 questionnaire, including 3 in the control group and 2 in the intervention group. The intervention group showed a slightly higher mean dependence score (mean 18.5, SD 7.8) than the control group (mean 14.7, SD 5.6).

### Intervention Acceptability

At follow-up, 9 participants in the intervention group completed a survey assessing the acceptability of the intervention. Table 3 shows the acceptability ratings of the faith-based smoking cessation intervention. Overall, the faith-based intervention was acceptable, with 33.3% (n=3) of participants rating it as *very acceptable* and 55.6% (n=5) rating it as *acceptable*. In terms of affective attitude, 22.2% (n=2) reported that they strongly liked the intervention, while 66.7% (n=6) reported that they liked it. One participant reported having no opinion, and no participants reported disliking the intervention.

**Table 3.** Self-reported intervention acceptability ratings at follow-up (n=9).

TFA^a^ construct, survey questions, and response	Values, n (%)
General acceptability	
How acceptable was the faith-based program?	
Very acceptable	3 (33.3)
Acceptable	5 (55.6)
No opinion	1 (11.1)
Unacceptable	0 (0)
Very unacceptable	0 (0)
Affective attitude	
Did you like or dislike the faith-based smoking cessation intervention?	
Strongly liked it	2 (22.2)
Liked it	6 (66.7)
No opinion	1 (11.1)
Disliked it	0 (0)
Strongly disliked it	0 (0)
Burden	
The effort required to participate in and read the daily messages	
No effort	3 (33.3)
Little effort	3 (33.3)
No opinion	0 (0)
A lot of effort	3 (33.3)
Huge effort	0 (0)
Perceived effectiveness	
The intervention helped me to quit or reduce smoking	
Strongly agree	2 (22.2)
Agree	4 (44.4)
No opinion	2 (22.2)
Disagree	1 (11.1)
Strongly disagree	0 (0)
Opportunity cost	
Reading the messages interfered with my other commitments and priorities	
Strongly agree	1 (11.1)
Agree	4 (44.4)
No opinion	2 (22.2)
Disagree	2 (22.2)
Strongly disagree	0 (0)

a TFA: Theoretical Framework of Acceptability.

The perceived burden of participation and reading the daily messages varied: 3 participants reported no effort, 3 participants reported little effort, and 3 participants reported that it required considerable effort. However, no participants reported experiencing a huge effort to read the messages. Regarding perceived effectiveness, 22.2% (2/9) of participants *strongly agreed* and 44.4% (4/9) *agreed* that the intervention helped them to quit or reduce smoking, while only one participant disagreed. Responses were mixed regarding the extent to which the intervention affected participants’ other commitments and responsibilities. One participant strongly agreed, and 4 participants agreed that reading the messages interfered with other commitments.

### Smoking Abstinence

Complete abstinence from all tobacco products, defined as no cigarettes and no waterpipe use in the previous 7 days, was calculated among all participants. Product-specific abstinence was calculated only among participants who reported using that product at baseline. Therefore, cigarette abstinence was calculated among participants who reported cigarette smoking at baseline, including cigarette-only and dual users. Waterpipe abstinence was calculated among participants who reported waterpipe use at baseline, including waterpipe-only and dual users.

Table 4 presents 7-day point prevalence abstinence outcomes descriptively. Complete abstinence from all tobacco products in both groups was 20.6% (7/34). The intervention group had a slightly higher complete abstinence rate (4/17, 23.5%) than the control group (3/17, 17.6%). Among the 32 participants who reported cigarette smoking at baseline, including cigarette-only and dual users, 8 (25%) reported cigarette abstinence at follow-up. Cigarette abstinence was higher in the intervention group than in the control group (5/16, 31.3% vs 3/16, 18.8%).

**Table 4.** Tobacco abstinence in the intervention and control groups at follow-up (N=34).

Outcome	Intervention, n/N (%)	Control, n/N (%)	Total, n/N (%)
Complete abstinence (cigarette and waterpipe)	4/17 (23.5)	3/17 (17.6)	7/34 (20.6)
Cigarette abstinence	5/16 (31.3)	3/16 (18.8)	8/32 (25.0)
Waterpipe abstinence	2/4 (50.0)	0/4 (0.0)	2/8 (25.0)

Among the 8 participants who reported waterpipe use at baseline, including 6 dual users and 2 waterpipe-only users, 2 (25%) reported waterpipe abstinence at follow-up. Both were baseline dual users in the intervention group. Neither of the 2 participants who reported waterpipe-only use at baseline reported abstinence from waterpipe smoking at follow-up. Given the small sample size, inferential statistical tests were not conducted.

### Religiosity

Table 5 presents multivariable binary regression analyses examining the relationship between each DUREL religiosity subscale and 7-day point prevalence abstinence, adjusted for treatment groups. For complete abstinence from cigarette and waterpipe smoking, ORA showed a positive but nonsignificant association with abstinence. However, there was no evidence of an association between NORA and complete abstinence (odds ratio 1.03, 95% CI 0.55‐1.91; *P*=.93), and IR was also not associated with complete abstinence (odds ratio 0.73, 95% CI 0.03‐18.71; *P=*.85).

**Table 5.** Multivariable binary logistic regression analyses of the associations Duke University Religion Index (DUREL) subscales and abstinence.

Outcome and predictor	Abstinent, n	Odds ratio (95% CI)	*P* value
Complete abstinence (cigarette and waterpipe) (N=34)			
ORA^a^	7	1.82 (0.96-3.46)	.07
NORA^b^	7	1.03 (0.55-1.91)	.93
IR^c^	7	0.73 (0.03-18.71)	.85
Cigarette abstinence (N=32)			
ORA	8	1.49 (0.88-2.52)	.14
NORA	8	1.20 (0.64-2.23)	.57
IR	8	0.83 (0.03-21.09)	.91

a ORA: organizational religious activity.

b NORA: nonorganizational religious activity.

c IR: intrinsic religiosity.

Among the 32 participants who reported cigarette smoking at baseline, the point estimate similarly suggested that higher ORA was associated with higher odds of cigarette abstinence, although the association was not statistically significant (odds ratio 1.49, 95% CI 0.88‐2.25; *P=*.14). There was no evidence of an association between NORA and IR with cigarette abstinence. Waterpipe abstinence was not included in the logistic regression analysis because only 8 participants reported waterpipe use at baseline, and only 2 of them reported waterpipe abstinence at follow-up.

### MTSS

Table 6 illustrates the distribution of participants across the 7 levels of the MTSS scale at baseline and follow-up. In the intervention group, 82.4% (14/17) of participants fell into the moderate to high motivation categories (levels 4‐7), reflecting either a desire to quit smoking without a clear plan or a definite intention to quit within 1 to 3 months. Only a small number of participants (3/17, 17.65%) reported low motivation to quit (levels 1‐2). At follow-up, the motivation levels remained largely unchanged in the intervention group. At baseline, the majority of participants in the control group (7/17, 41.2%) were at level 5 (*I want to quit smoking and I hope to soon),* followed by 5 out of 17 (29.4%) participants at level 4 *(I really want to quit smoking, but I have not thought about when*). This suggests that participants in the control group had a desire to quit but did not have a clear plan to do so soon. The pattern remained unchanged at follow-up, with the majority still in levels 4 and 5. Overall, both groups showed moderate to high motivation to quit smoking. However, the intervention group had a greater proportion of participants in the higher motivation categories, particularly levels 6 and 7, compared to the control group.

**Table 6.** Motivation to quit smoking at baseline and follow-up (N=34).

MTSS^a^ level	MTSS item	Intervention (n=17), n (%)		Control (n=17), n (%)	
		Baseline	Follow-up	Baseline	Follow-up
1	I do not want to quit smoking	1 (5.9)	1 (5.9)	0 (0)	0 (0)
2	I think I should quit smoking, but I do not really want to	1 (5.9)	1 (5.9)	2 (11.8)	2 (11.8)
3	I want to quit smoking, but I have not thought about when	1 (5.9)	1 (5.9)	1 (5.9)	1 (5.9)
4	I REALLY want to quit smoking, but I don’t know when I will	4 (23.5)	3 (17.6)	5 (29.4)	4 (23.5)
5	I want to quit smoking, and I hope to soon	4 (23.5)	4 (23.5)	7 (41.2)	6 (35.3)
6	I REALLY want to quit smoking and intend to in the next 3 months	1 (5.9)	2 (11.8)	0 (0)	2 (11.8)
7	I REALLY want to quit smoking and intend to in the next month	5 (29.4)	5 (29.4)	2 (11.8)	2 (11.8)

a MTSS: motivation to stop smoking.

### Quit Attempts

Table 7 presents quit attempts at baseline and follow-up for both groups. At baseline, among 17 participants, 11 (64.7%) in the intervention group and 9 (52.9%) in the control group reported at least one quit attempt in the 30 days. At follow-up, quit attempts increased slightly in both groups, reaching 13 (76.5%) in the intervention group and 10 (58.8%) in the control group. Within-group changes from baseline to follow-up were not statistically significant in either the intervention group or the control group. Both groups showed a small increase in quit attempts from baseline to follow-up, with a slightly larger absolute increase in the intervention group than in the control group (11.8 vs 5.9).

**Table 7.** Quit attempts at baseline and follow-up in the intervention (n=17) and control (n=17) groups.

Group	Baseline quit attempts, n (%)		Follow-up quit attempts, n (%)	Change in percentage point	Within-group *P* value
Intervention	11 (64.7)		13 (76.5)	+11.8	.50
Control	9 (52.9)		10 (58.8)	+5.9	>.99

## Discussion

### Principal Findings

This is the first pilot RCT to evaluate the feasibility and acceptability of an Islamic faith-based smoking cessation intervention delivered via WhatsApp messages in Saudi Arabia, contributing to the literature on both faith-based approaches to smoking cessation among Muslims and digital delivery methods in this context. Overall, the findings indicate high acceptability and positive attitudes toward the intervention, while also highlighting areas for improvement, particularly in relation to burden and opportunity costs. These findings are consistent with research among Somali Muslim men in the United States, who reported high acceptability and perceived helpfulness of religiously tailored smoking cessation messages [45].

The study successfully recruited 34 adult Muslim smokers, achieving 68% of the target sample of 50 participants. At follow-up, 22 of the 34 randomized participants completed the survey, resulting in an overall retention rate of 64.7%. Among the 22 participants who returned the survey, 7 (31.8%) participants did not complete all survey items. Both recruitment and retention rates fall within ranges supporting progression to a definitive trial following necessary modifications [40]. These findings suggest that a full-scale trial may be feasible; however, substantial improvements in recruitment, participant retention, and follow-up data collection would be required before proceeding.

A total of 44 WhatsApp messages were sent over the 21-day intervention period. These included 1 welcome message, 2 messages sent daily, and 1 farewell message, demonstrating the practicality of delivering a smoking cessation intervention via WhatsApp. This aligns with previous research demonstrating the feasibility of recruiting 50 Muslim Somali men and delivering 78 religiously tailored text messages, at a rate of 2 per day, over 5 weeks [45].

Self-reported satisfaction with the faith-based intervention was generally positive. Participants found the messages helpful in supporting their efforts to quit or reduce smoking. However, the perceived burden and effort associated with reading the daily messages varied. While some participants reported no effort, others indicated that reading the messages required either little effort or a great deal of effort. In terms of opportunity cost, 5 participants reported that the intervention interfered with other daily commitments. Future refinements could include shortening the messages to reduce burden and opportunity costs.

We found that participants in both groups had relatively high nicotine dependence at baseline as measured by the FTND, with slightly higher scores observed in the control group compared to the intervention group. Both groups reported moderate to high levels of waterpipe nicotine dependence, measured by the LWDS-11, with the intervention group scoring higher than the control group. Previous research has shown that higher nicotine dependence is associated with failure of quit attempts, while lower levels of nicotine dependence predict successful quit attempts [46]. One possible explanation for the high FTND scores observed in this study is that participants were recruited from an antismoking clinic, where individuals are often more nicotine dependent and more likely to seek professional support and treatment. Individuals who smoke and seek help from specialist smoking cessation clinics tend to have higher nicotine dependence compared to those from the general population [47]. Future research could recruit participants from community settings, such as workplaces, faith-based organizations, cafes, social media platforms, and social networks, to obtain a sample with varying levels of nicotine dependence.

The intervention group reported slightly higher rates of complete abstinence from all tobacco products and cigarette abstinence compared with the control group. Complete abstinence from all tobacco products was reported by 23.5% (4/17) of participants in the intervention group and 17.6% (3/17) in the control group. Cigarette abstinence among baseline cigarette users, including cigarette-only and dual users, was also higher in the intervention group (5/16, 31.3%) than in the control group (3/16, 18.8%). These findings are descriptive and should be interpreted with caution, given the small sample size.

Waterpipe abstinence was recalculated among participants who reported using waterpipe at baseline, including dual users and waterpipe-only users. Among these participants, waterpipe abstinence was reported only in the intervention group and only among baseline dual users. Although these findings are preliminary, they indicate that the intervention may have some relevance for dual users of cigarettes and waterpipe. This is important because the dual use of cigarettes and waterpipe is common in many Arab countries [48]. Evidence suggests that the dual use is associated with higher nicotine dependence [49], which may lower the chances of successful cessation. Therefore, researchers have emphasized the need for comprehensive strategies to address dual and multiple tobacco product use [50].

More than half of participants in both groups reported at least one quit attempt at baseline and follow-up. Quit attempts slightly increased in both groups over the intervention period, with a larger absolute increase in the intervention group than in the control group. However, these findings should be treated as descriptive and exploratory. Furthermore, restricting the definition of quit attempts to abstinence lasting at least 24 hours may underestimate the number of quit attempts and miss true quit attempts that did not last for 24 hours [51].

Religiosity did not significantly predict cessation outcomes in this pilot study. Logistic regression analyses adjusted for treatment group were used to examine whether DUREL religiosity subscales were associated with abstinence outcomes. ORA, measured by frequency of religious attendance, showed a positive but nonsignificant association with 7-day complete abstinence from all forms of tobacco, cigarettes and waterpipe, and cigarette-only abstinence. Although this association was not statistically significant, it may suggest that more frequent religious attendance supports tobacco cessation and warrants further exploration in research. Possible mechanisms for this relationship can be understood through socialization and control perspectives [52]. For example, attending mosques and religious activities exposes individuals to moral teachings that discourage harmful behaviors such as smoking. Social monitoring and support within religious communities and attending mosques and religious events provide opportunities for accountability and support from peers, which may encourage cessation and reduce the likelihood of relapse. Faith-based smoking cessation interventions might achieve greater impact if they leveraged mosque-based interventions and group spiritual supports. For example, a London-wide Ramadan campaign implemented in mosques was effective in raising awareness of local smoking cessation services and encouraging smokers to make a quit attempt [53]. Furthermore, delivering health promotion messages during Friday sermons in mosques can be highly acceptable and desirable [54], highlighting the potential of mosque-based interventions as venues to leverage organizational religiosity.

While previous research suggests that private or personal religious practices, such as Quran recitation, may lead to a significant reduction in smoking intensity and cravings and may support cessation [55], we found no association between private nonorganizational religious practices and IR with cessation. The IR subscale measures the extent to which religious beliefs guide daily life [39]. Research has shown that smoking is inversely associated with Muslim IR, such that individuals with higher levels of IR were less likely to smoke and perceived smoking as conflicting with their religious beliefs [56]. Because smoking is considered forbidden in Islam, Muslims with higher IR are more likely to view smoking as incompatible with their religious beliefs.

This study has several strengths. First, the intervention was informed by qualitative research with the target population and co-developed with religious scholars, which enhanced its cultural relevance and religious appropriateness. Second, we measured religiosity at baseline using a brief, multidimensional scale, which allowed for a nuanced understanding of how different aspects of religiosity may influence smoking behaviors among Muslims [57]. Third, the use of a two-arm study design comprising an intervention group and a comparison group allowed us to discern the effects of the religiously tailored messages from smoking-related health messages.

There are several limitations. First, a priori progression criteria for recruitment and retention were not specified. Therefore, these outcomes should be interpreted descriptively rather than as evidence that a predetermined threshold for progression was met. Nevertheless, the recruitment rate of 68% (34/50) and the retention rate of 64.7% (22/34) indicate that a future definitive trial may be feasible following necessary modifications to improve recruitment and retention. Second, the study was not adequately powered to detect a cessation outcome and did not achieve the targeted sample size of 25 participants per group, possibly because recruitment was limited to a single clinic, which restricted the pool of eligible participants, who may not be representative of other Muslims in other regions of Saudi Arabia or internationally. Third, participants in the control group were aware that they would receive smoking-related health messages only, which may have influenced their behavior in the trial and responses to outcome measures. Fourth, the intervention and follow-up periods were of short duration, limiting the ability to assess long-term abstinence beyond the end of the intervention, which is crucial to evaluate the long-term effects of an intervention [42]. However, while 6 months postquit date or postintervention initiation is considered the gold standard for measuring abstinence in cessation trials, a shorter duration may be appropriate in pilot feasibility studies [42]. Fifth, it could be that some participants had no interest in behavioral interventions and may have preferred pharmacotherapy such as nicotine replacement therapy [58]. In Saudi Arabia, awareness of behavioral therapies for smoking cessation is limited among individuals attending smoking cessation clinics [59]. Finally, we did not perform biochemical verification of self-reported smoking abstinence, which may have led to the overestimation of quit rates. However, as a pilot trial, self-reported abstinence was considered adequate. Furthermore, cultural sensitivities (eg, privacy concerns) and cost limitations were barriers to collecting biological samples.

### Conclusion

Delivering faith-based messages via WhatsApp was feasible and acceptable among Muslim adults who smoke in Saudi Arabia. However, challenges related to recruitment, retention, message burden, missing data, and retrospective trial registration limit the strength of the evidence. Given the small sample size and limited follow-up completion, smoking cessation and reduction outcomes should be interpreted as preliminary. Despite these limitations, the study provides important lessons for developing and implementing faith-based smoking cessation interventions. Future studies should recruit larger and more diverse samples across multiple smoking cessation clinics and community settings to strengthen the generalizability of findings and provide more robust evidence on the acceptability and effectiveness of the intervention.

## Supplementary material

10.2196/88796 Multimedia Appendix 1 Examples of messages for the intervention and control groups.

10.2196/88796 Checklist 1 CONSORT-EHEALTH Checklist

## Abbreviations

CONSORT: Consolidated Standards of Reporting Trials
DUREL: Duke University Religion Index
FTND: Fagerström Test for Nicotine Dependence
IR: intrinsic religiosity
LWDS: Lebanon Waterpipe Dependence Scale
MTSS: motivation to stop smoking
NORA: nonorganizational religious activity
ORA: organizational religious activity
RCT: randomized controlled trial
TFA: Theoretical Framework of Acceptability
WHO: World Health Organization
