# Supplementary material for: Acceptability and Feasibility of an Islamic Faith-Based Smoking Cessation Intervention Delivered via WhatsApp for Muslim Smokers in Saudi Arabia: Pilot Randomized Controlled Trial
Source: JMIR Form Res. 2026 Oct 15;10:e88796. doi: 10.2196/88796 (PMC13671676; doi:10.2196/88796)
Supplement: Supplementary file 2 [file formative-v10-e88796-s001.docx]

## Appendix. Examples of messages for the intervention and control groups

| **Messages for the intervention group** | |
| --- | --- |
| **Message in English** | **Message in Arabic** |
| [Enough Smoking] Social support is very important when quitting smoking. Tell your family and friends about your journey to quit and ask them to remind you of your goals. This is considered an act of cooperation in righteousness and piety. Allah Almighty says: *“And cooperate with one another in righteousness and piety.”* | [كفى تدخين] الدعم الاجتماعي مهم جدًا عند الإقلاع عن التدخين. أخبر عائلتك و أصدقائك عن رحلتك في الإقلاع عن التدخين. اطلب منهم تذكيرك بأهدافك في الإقلاع عن التدخين. وهذا يعتبر هذا تعاونا على البر والتقوى. **يقول الله تعالى: وَتَعَاوَنُوا عَلَى الْبِرِّ وَالتَّقْوَىٰ** |
| [Enough Smoking] If you experience anxiety, irritability, or difficulty focusing and sleeping, these are common symptoms of nicotine withdrawal. Do not be discouraged. These symptoms will disappear over time. Rely on patience and prayer for strength, as Allah Almighty says: *“O you who have believed, seek help through patience and prayer. Indeed, Allah is with the patient.”* | [كفى تدخين] إذا كنت تشعر بالقلق وسرعة الغضب وتعاني من صعوبة في التركيز و النوم فقد تكون هذه أعراض انسحاب النيكوتين. لاتقلق ، فوجود أعراض الانسحاب أمر طبيعي عند الإقلاع عن التدخين وسوف تختفي الأعراض مع مرور الوقت. استعن بالصبر والصلاة كما **قال تعالى: يَا أَيُّهَا الَّذِينَ آمَنُوا اسْتَعِينُوا بِالصَّبْرِ وَالصَّلَاةِ ۚ إِنَّ اللَّهَ مَعَ الصَّابِرِينَ** |
| [Enough Smoking] Islam permits all that preserves life, intellect, and the body, and prohibits all that causes them harm. The majority of scholars have ruled that smoking is forbidden because it harms the body. Allah Almighty says: *“He allows them as lawful what is good and pure and prohibits them from what is foul and harmful.”* | [كفى تدخين] أحل الإسلام كل ما يحافظ على النفس والعقل والجسد، وحرم كل ما يضر النفس والعقل والجسد. وقد ذهب جمهور العلماء إلى أن التدخين محرم لأنه مضر للجسم. **يقول تبارك وتعالى: وَيُحِلُّ لَهُمُ الطَّيِّبَاتِ وَيُحَرِّمُ عَلَيْهِمُ الْخَبَائِثَ** |
| [Enough Smoking] Your smoking harms those around you. Studies have shown that people exposed to second-hand smoke by sitting with smokers are at risk of developing cancer and respiratory diseases. Remember the saying of our Prophet ﷺ: *“A Muslim is the one from whose tongue and hand other Muslims are safe.”* | [كفى تدخين] تدخينك يؤذي الأشخاص من حولك. أثبتت الدراسات أن الذين يتعرضون لانبعاثات الدخان من خلال الجلوس مع المدخنين معرضون لخطر الإصابة بالسرطان وأمراض الجهاز التنفسي. **تذكر قول رسولنا صلى الله عليه وسلم: "المسلم من سلم المسلمون من لسانه ويده".** |
| **Messages for the control group** | |
| **Message in English** | **Message in Arabic** |
| [Enough Smoking] Social support is very important when quitting smoking. Tell your family and friends about your journey to quit and ask them to remind you of your goals | [كفى تدخين] الدعم الاجتماعي مهم جدًا عند الإقلاع عن التدخين. أخبر عائلتك و أصدقائك عن رحلتك في الإقلاع عن التدخين. اطلب منهم تذكيرك بأهدافك في الإقلاع عن التدخين |
| [Enough Smoking] If you experience anxiety, irritability, or difficulty focusing and sleeping, these are common symptoms of nicotine withdrawal. Do not be discouraged. These symptoms will disappear over time. | [كفى تدخين] إذا كنت تشعر بالقلق وسرعة الغضب وتعاني من صعوبة في التركيز و النوم فقد تكون هذه أعراض انسحاب النيكوتين. لاتقلق ، فوجود أعراض الانسحاب أمر طبيعي عند الإقلاع عن التدخين وسوف تختفي الأعراض مع مرور الوقت. |
| [Enough Smoking] Smoking causes oral cancer, gum disease, and other diseases of the mouth and teeth. It also causes very bad breath. Quit smoking to protect the health of your mouth and teeth. | [كفى تدخين] التدخين يسبب سرطان الفم وأمراض اللثة وغيرها من أمراض الفم والأسنان. كما يسبب التدخين رائحة كريهة جدا للفم. أقلع عن التدخين للحفاظ على صحة فمك وأسنانك. |
| [Enough Smoking] Your smoking harms those around you. Studies have shown that people exposed to second-hand smoke by sitting with smokers are at risk of developing cancer and respiratory diseases. | [كفى تدخين] تدخينك يؤذي الأشخاص من حولك. أثبتت الدراسات أن الذين يتعرضون لانبعاثات الدخان من خلال الجلوس مع المدخنين معرضون لخطر الإصابة بالسرطان وأمراض الجهاز التنفسي. |
